# The Design of a Frame-Like ZnO FBAR Sensor for Achieving Uniform Mass Sensitivity Distributions

**DOI:** 10.3390/s20082408

**Published:** 2020-04-23

**Authors:** Xueli Zhao, Zinan Zhao, Bin Wang, Zhenghua Qian, Tingfeng Ma

**Affiliations:** 1 State Key Laboratory of Mechanics and Control of Mechanical Structures, College of Aerospace Engineering, Nanjing University of Aeronautics and Astronautics, Nanjing 210016, China; zxl975327@nuaa.edu.cn (X.Z.); zinan_zhao@163.com (Z.Z.); wangbin1982@nuaa.edu.cn (B.W.); 2 Piezoelectric Device Laboratory, School of Mechanical Engineering and Mechanics, Ningbo University, Ningbo 315211, China; matingfeng@nbu.edu.cn

**Keywords:** FBAR mass sensor, frame-like driving electrode, thickness-extensional mode, uniform mass sensitivity distribution

## Abstract

In this paper, an infinite circular ZnO thin film bulk acoustic resonator (FBAR) with a frame-like electrode operating at the thickness-extensional (TE) mode is studied. Two-dimensional scalar differential equations established for the problem in the Cartesian coordinate system are successfully solved by transforming them into normal Bessel equations and modified Bessel equations in the cylindrical coordinate system. Resonant frequencies and vibration distributions are obtained for this frame-like FBAR sensor. A nearly uniform mass sensitivity distribution in the active area is achieved by designing proper electrode size and mass ratio of the driving electrode to the ZnO film. Numerical results show that compared with the reported ring electrode FBAR sensor, the novel frame-like electrode FBAR can achieve a maximum optimization ratio (up to 97.90%) on the uniformity of the mass sensitivity distribution in the active area under the same structural parameters, which is also higher than the optimization ratio 77.63% obtained by the reported double-ring electrode design. Moreover, the mechanism to achieve a very uniform mass sensitivity distribution in the active area by the frame-like electrode is explained in detail according to dispersion curves. Namely, when the resonant frequency of the FBAR sensor is close to the cut-off frequency of the active region in the dispersion curve, the mass sensitivity distribution is nearly uniform. These conclusions provide a theoretical guidance for the design and optimization of ZnO FBAR mass sensors with high performance.

## 1. Introduction

In the past decades, since the relationship between the frequency changes of a quartz crystal and the mass added to its surface was discovered [1], quartz crystal microbalances (QCMs) based on quartz crystal resonators (QCRs) have been widely used in the field of mass sensing [2,3,4,5]. However, since the thickness of a quartz crystal plate is usually in the order of millimeter, a QCR usually operates at a frequency of megahertz, which limits the mass sensitivity of QCMs [6,7]. At present, piezoelectric thin film bulk acoustic resonators (FBARs) made from ZnO or AlN film have been proposed and applied in telecommunication field. As a result of its small size in thickness direction, FBARs can operate at an ultra-high frequency of gigahertz, and hence the FBAR sensors can achieve a higher mass sensitivity than traditional QCMs [8,9]. 

In addition to higher mass sensitivity, uniform mass sensitivity distribution is also a key influence factor to improve the performance of the sensors [10,11,12,13]. The working principle of mass sensors is to detect the frequency changes of the sensor induced by the added mass deposited on the sensor’s surface. The relation between frequency shifts and the added mass has the relation as [10]

(1) $$\Delta f=-S_{f}(r,\theta )\Delta m$$

where Δf is the frequency change and Δm is the added mass [14,15]. $S_{f}(r,\theta )$ is the mass sensitivity function, which is written as [10]

(2) $$S_{f}(r,\theta )=\frac{|f^{n}(r,\theta ){|}^{2}}{2\pi \int_{0}^{\infty }r|f^{n}(r,\theta ){|}^{2}dr}\cdot C_{f}$$

where $f^{n}(r,\theta )$ is the displacement distribution function of the sensor.$C_{f}$ is the Sauerbrey’s sensitivity constant. It can be seen from Equation (1) that if $S_{f}(r,\theta )$ is uniform, the frequency shifts of the mass sensor only depend on the added mass. Otherwise, the frequency shifts are also dependent on $S_{f}(r,\theta )$, which is a function of the position of the added mass. Therefore, it is necessary to achieve the uniform mass sensitivity distribution function $S_{f}(r,\theta )$. However, for typical QCMs and FBARs, the mass sensitivity distribution is usually quite non-uniform and has a bell-shaped contour in the active area [16,17]. Therefore, many works were carried out to achieve more uniform mass sensitivity distributions in QCM and FBAR sensors. For example, in QCMs some methods by optimizing the electrode geometry to obtain flatter mass sensitivity curve were proposed, such as the “n-m” electrodes [10], ring [18], dot-ring, and double-ring electrodes [11]. Similarly, some researchers also made some positive explorations in FBAR mass sensors. Zhao et al. optimized the parameters of the rectangular ring electrodes in a ZnO FBAR sensor by using the Ritz method based on the two-dimensional scalar equations, and obtained a relatively flat displacement curve by adjusting the electrode size and mass ratio in detail [19]. Moreover, Liu et al. studied the displacement distribution of a circular ring electrode FBARs by analytical solution and discovered the effects of electrode size and inertia on the displacement distributions [20]. However, the ring electrode FBARs, whether circular or rectangular, can only produce bimodal distributions in the electrode region, which means that the mass sensitivity curves cannot satisfy the actual requirement, i.e., very uniform mass sensitivity distributions. Therefore, it is necessary to propose a new structure to achieve a more uniform mass sensitivity distribution. 

In this paper, a frame-like electrode FBAR is proposed to obtain a more uniform mass sensitivity distribution. The thickness-extensional (TE) mode of a circular frame-like electrode FBAR is studied theoretically based on the two-dimensional scalar equations derived by Tiersten and Stevens [21]. According to the coordinate transformation, the two-dimensional scalar equations in the Cartesian coordinate system are transformed to normal Bessel equations and modified Bessel equation in cylindrical coordinate system. Meanwhile, a very uniform mass sensitivity distribution is achieved by optimizing the electrode size and mass ratio. The mechanism to achieve the very uniform mass sensitivity distribution in the active area by the frame-like electrode is also discussed by studying the dispersion curves in different areas of the FBAR sensor. These results can provide a fundamental reference to the structural design and optimization of FBAR mass sensors.

## 2. Governing Equations

**Figure 1:**
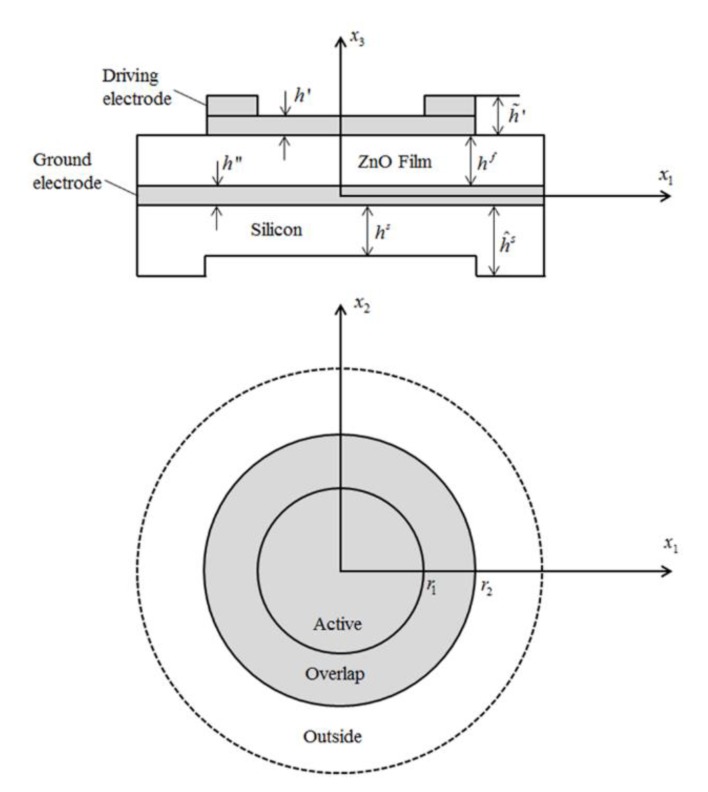
The schematic of a ZnO film bulk acoustic resonator (FBAR) with a circular frame-like driving electrode. It consists of a driving electrode, a ZnO film, a ground electrode, and a Si substrate, where *h* indicates the thickness of each region and *r* represents the electrode size.

This paper considers a ZnO FBAR mass sensor with its top surface covered by a circular frame-like electrode, as shown in Figure 1. For the ZnO FBAR, the main operating mode is TE mode. Considering the time-harmonic free vibration at a resonant frequency ω in this FBAR sensor, the main displacement component of the *n*th thickness-extensional mode can be approximately written as [21]

(3) $$u_{3}^{n}≅f^{n}(x_{1},x_{2})g^{n}(x_{3})e^{i\omega t}$$

where $g^{n}(x_{3})$ is the displacement variations of the TE mode in the $x_{3}$ direction. The in-plane displacement variations of the TE mode is described by $f^{n}(x_{1},x_{2})$, governed by the two-dimensional scalar differential equations derived by Tiersten and Stevens [21]. These scalar equations are slightly different for the electroded area and the unelectroded area.

In the outside area without a driving electrode, from Equation (6.2) of [21], the governing equation is

(4) $$M_{n}\left(\frac{\partial^{2}f^{n}}{\partial x_{1}^{2}}+\frac{\partial^{2}f^{n}}{\partial x_{2}^{2}}\right)-{\bar{c}}_{33}^{f}{\hat{\eta }}_{f^{n}}^{2}f^{n}+\rho^{f}\omega^{2}f^{n}=0$$

In the overlap area with the thicker driving electrode, $f^{n}(x_{1},x_{2})$ is governed by

(5) $$M_{n}\left(\frac{\partial^{2}f^{n}}{\partial x_{1}^{2}}+\frac{\partial^{2}f^{n}}{\partial x_{2}^{2}}\right)-{\bar{c}}_{33}^{f}{\tilde{\eta }}_{f^{n}}^{2}f^{n}+\rho^{f}\omega^{2}f^{n}=0$$

Similarly, in the active area with a thinner driving electrode, the governing equation is

(6) $$M_{n}\left(\frac{\partial^{2}f^{n}}{\partial x_{1}^{2}}+\frac{\partial^{2}f^{n}}{\partial x_{2}^{2}}\right)-{\bar{c}}_{33}^{f}{\bar{\eta }}_{f^{n}}^{2}f^{n}+\rho^{f}\omega^{2}f^{n}=0$$

where $\rho^{f}$ is the mass density of the thin film and ω is the resonant frequency. $\eta_{f}$ is the acoustic wavenumber of TE vibration, which is distinct under different electrode coverings. ${\bar{c}}_{33}^{f}$ is related to the material constants of thin film. *M_n_* is determined by the material constants of the electrodes, thin film and Si substance. The calculations of them are given in the appendix of [16].

For a convenient simulation, we transform the Cartesian coordinate system into the cylindrical coordinate system. Introducing the coordinate transformation

(7) $$x_{1}=r\cos m\theta ,\;x_{2}=r\sin m\theta$$

and denoting

(8) $${\bar{\omega }}_{n}^{2}=\frac{{\bar{c}}^{f}{}_{33}{\bar{\eta }}^{2}{}_{f^{n}}}{\rho^{f}},\;{\tilde{\omega }}_{n}^{2}=\frac{{\bar{c}}^{f}{}_{33}{\tilde{\eta }}^{2}{}_{f^{n}}}{\rho^{f}},\;{\hat{\omega }}_{n}^{2}=\frac{{\bar{c}}^{f}{}_{33}{\hat{\eta }}^{2}{}_{f^{n}}}{\rho^{f}}$$

then Equations (4)–(6) can be written as

(9) $$\begin{array}{l} M_{n}\left(\frac{\partial^{2}f^{n}}{\partial r^{2}}+\frac{1}{r}\frac{\partial f^{n}}{\partial r}-\frac{m^{2}}{r^{2}}f^{n}\right)+[\rho^{f}（\omega^{2}-{\bar{\omega }}_{n}{}^{2})]f^{n}=0,\;r<r_{1}\\ M_{n}\left(\frac{\partial^{2}f^{n}}{\partial r^{2}}+\frac{1}{r}\frac{\partial f^{n}}{\partial r}-\frac{m^{2}}{r^{2}}f^{n}\right)+[\rho^{f}（\omega^{2}-{\tilde{\omega }}_{n}{}^{2})]f^{n}=0,\;r_{1}\leq r\leq r_{2}\\ M_{n}\left(\frac{\partial^{2}f^{n}}{\partial r^{2}}+\frac{1}{r}\frac{\partial f^{n}}{\partial r}-\frac{m^{2}}{r^{2}}f^{n}\right)+[\rho^{f}（\omega^{2}-{\hat{\omega }}_{n}{}^{2})]f^{n}=0,\;r_{2}<r<\infty \end{array}$$

Then denoting

(10) $$\begin{array}{l} {\bar{\alpha }}^{2}=\frac{\rho^{f}(\omega^{2}-{\bar{\omega }}_{n}{}^{2})}{M_{n}}>0\\ {\tilde{\alpha }}^{2}=\frac{\rho^{f}(\omega^{2}-{\tilde{\omega }}_{n}{}^{2})}{M_{n}}>0\\ {\hat{\alpha }}^{2}=\frac{\rho^{f}({\hat{\omega }}_{n}{}^{2}-\omega^{2})}{M_{n}}>0 \end{array}$$

we obtain the following three equations in form of Bessel equation and modified Bessel equation

(11) $$\begin{array}{l} \left(\frac{\partial^{2}f^{n}}{\partial r^{2}}+\frac{1}{r}\frac{\partial f^{n}}{\partial r}\right)+\left({\bar{\alpha }}^{2}-\frac{m^{2}}{r^{2}}\right)f^{n}=0,\;r<r_{1}\\ \left(\frac{\partial^{2}f^{n}}{\partial r^{2}}+\frac{1}{r}\frac{\partial f^{n}}{\partial r}\right)+\left({\tilde{\alpha }}^{2}-\frac{m^{2}}{r^{2}}\right)f^{n}=0,\;r_{1}\leq r\leq r_{2}\\ \left(\frac{\partial^{2}f^{n}}{\partial r^{2}}+\frac{1}{r}\frac{\partial f^{n}}{\partial r}\right)+\left(-{\hat{\alpha }}^{2}-\frac{m^{2}}{r^{2}}\right)f^{n}=0,\;r_{2}<r<\infty \end{array}$$

Further, we introduce

(12) $$\begin{array}{l} \xi_{1}=\bar{\alpha }r,\;r<r_{1}\\ \xi_{2}=\tilde{\alpha }r,\;r_{1}\leq r\leq r_{2}\\ \eta =\hat{\alpha }r,\;r_{2}<r<\infty \end{array}$$

and then Equation (11) can be written as the following standard Bessel equations and modified Bessel equation

(13) $$\begin{array}{l} \frac{\partial^{2}f^{n}}{\partial \xi_{1}{}^{2}}+\frac{1}{\xi_{1}}\frac{\partial f^{n}}{\partial \xi_{1}}+\left(1-\frac{m^{2}}{\xi_{1}{}^{2}}\right)f^{n}=0,\;r<r_{1}\\ \frac{\partial^{2}f^{n}}{\partial \xi_{2}{}^{2}}+\frac{1}{\xi_{2}}\frac{\partial f^{n}}{\partial \xi_{2}}+\left(1-\frac{m^{2}}{\xi_{2}{}^{2}}\right)f^{n}=0,\;r_{1}\leq r\leq r_{2}\\ \frac{\partial^{2}f^{n}}{\partial \eta^{2}}+\frac{1}{\eta }\frac{\partial f^{n}}{\partial \eta }-\left(1+\frac{m^{2}}{\eta^{2}}\right)f^{n}=0,\;r_{2}<r<\infty \end{array}$$

## 3. Analytical Solution

Considering the infinite ZnO FBAR mass sensor in Figure 1, the corresponding continuity conditions and boundary conditions are separately

(14a) $$f^{n}\;\mathrm{is}\;\mathrm{finite},\;r=0$$

(14b) $$f^{n}(r_{1}{}^{-})=f^{n}(r_{1}{}^{+}),\;{\frac{df^{n}}{dr}|}_{r_{1}{}^{-}}={\frac{df^{n}}{dr}|}_{r_{1}{}^{+}}$$

(14c) $$f^{n}(r_{2}{}^{-})=f^{n}(r_{2}{}^{+}),{\;\frac{df^{n}}{dr}|}_{r_{2}{}^{-}}={\frac{df^{n}}{dr}|}_{r_{2}{}^{+}}$$

(14d) $$f^{n}\to 0,\;r\to \infty$$

The general solutions of the Bessel equation and modified Bessel equation in Equation (13) satisfying Equation (14a) and (14d) can be written as

(15) $$f^{n}=\left\{ \begin{array}{l} AJ_{m}(\bar{\alpha }r),\;r<r_{1}\\ BJ_{m}(\tilde{\alpha }r)+CY_{m}(\tilde{\alpha }r),\;r_{1}\leq r\leq r_{2}\\ DK_{m}(\hat{\alpha }r),\;r_{2}<r<\infty \end{array}\right.$$

where *A*, *B*, *C,* and *D* are undetermined constants. *J_m_* and *Y_m_* are the *m*th-order Bessel function of the first kind and second kind, respectively. *K_m_* is the *m*th-order modified Bessel function of the second kind. Substitution of Equation (15) into the continuity conditions in Equation (14b) and (14c) gives four linear homogeneous equations for the undetermined constants *A*, *B*, *C,* and *D*. For the existence of nontrivial solutions, the determinant of the coefficient matrix of the equations has to vanish, which gives the frequency equation. The nontrivial solutions of *A*, *B*, *C,* and *D* determine the corresponding vibration modes. Then substituting Equation (15) into Equation (2), we can obtain the mass sensitivity curves of the frame-like electrode FBAR.

## 4. Numerical Examples and Discussions

As a numerical example, we consider a circular infinite ZnO FBAR mass sensor with a frame-like driving electrode in Figure 1 operating at the fundamental TE mode with *n* = 1 and *m* = 0. The thickness of the ZnO film is chosen as $h^{f}=15\;\mu m$ and the substrate layer thickness is fixed as $h^{s}={\hat{h}}^{s}=5\;\mu m$. The mass ratio between the ground electrode and the film is fixed to be R’’=0.0453. The material constants of ZnO thin film, Si substrate, and Au electrodes can be found in [22].

### 4.1. Validity of the Novel Frame-Like Electrode Design

In this section, we aim to check the validity of the novel frame-like electrode designing in a ZnO FBAR mass sensor through comparing the mass sensitivity distributions of a normal circular electrode FBAR and a ring electrode FBAR with those of a frame-like electrode FBAR. For the normal circular FBAR, the mass ratio of the driving electrode to the film is selected as R’=0.01, and the radius of the circular electrode is r=60 μm. For the frame-like electrode FBAR, the mass ratio of the active driving electrode to the ZnO thin film is equal to the mass ratio R’ of the normal circular FBAR, but in the overlap area of the frame-like electrode FBAR $\tilde{R}’$ is equal to 0.04, which is the same as the mass ratio in the ring electrode FBAR. According to $\tilde{R}’=(\rho ’\tilde{h}’)/(\rho^{f}h^{f})$, the value of $\tilde{R}’$ can be simply achieved by altering the overlap thickness $\tilde{h}’$. In addition, the electrode radii of the active and overlap areas are $r_{1}=20\;\mu m$ and $r_{2}=60\;\mu m$, respectively.

**Figure 2:**
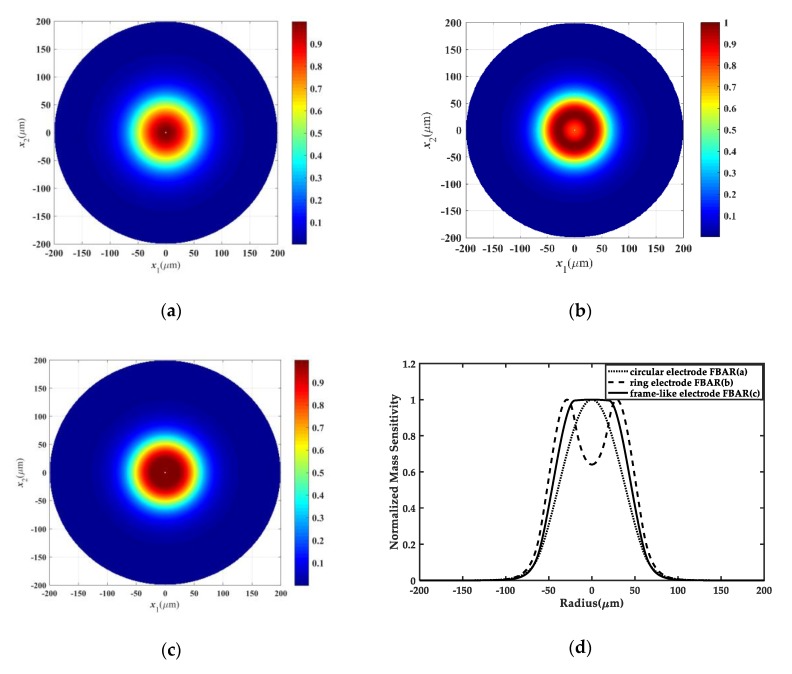
2D top view of the fundamental thickness-extensional (TE) mode in the normal circular electrode (energy-trapped) FBAR sensor (**a**), the ring electrode FBAR sensor (**b**), and the frame-like electrode FBAR sensor (**c**). Normalized mass sensitivity distributions of these three cases can be seen in (**d**).

Figure 2a–c illustrate the TE vibration modes in the normal circular electrode FBAR, the ring electrode FBAR and the frame-like electrode FBAR, respectively. Figure 2d shows the normalized mass sensitivity distributions of these three cases. Obviously, compared with the bell-shaped mass sensitivity curve of the normal circular electrode FBAR and the bimodal distribution of the ring electrode FBAR, the frame-like electrode FBAR can produce a nearly uniform mass sensitivity distribution in the active area, which is desirable in real mass sensor applications.

### 4.2. Effect of Structural Parameters of the Frame-Like Electrode

In Section 4.1, we have concluded that FBAR with the frame-like electrode can obtain a nearly uniform mass sensitivity distribution in the active region. In this section, we further study the influence of the structural size of the frame-like electrode and the mass ratio $\tilde{R}’$ to achieve more uniform mass sensitivity distributions, respectively. The values of the structural parameters are the same as those used in Section 4.1 except for $r_{1}$,$r_{2}$, and $\tilde{R}’$. These three parameters will be given in later specific analysis. 

**Figure 3:**
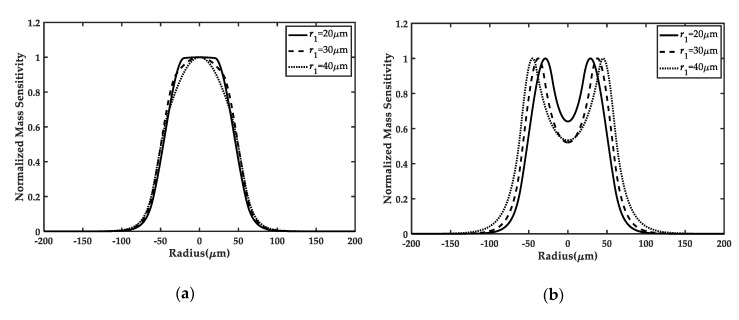
Comparison of the frame-like electrode FBAR (**a**) and the ring electrode FBAR (**b**) [20] for different $r_{1}$, when $r_{2}$ is fixed as 60 μm and $\tilde{R}’$ is equal to 0.04. With the decreasing of $r_{1}$, the mass sensitivity distributions in the active region for the frame-like electrode become more uniform, but the distributions for the ring electrode are still concave.

**Figure 4:**
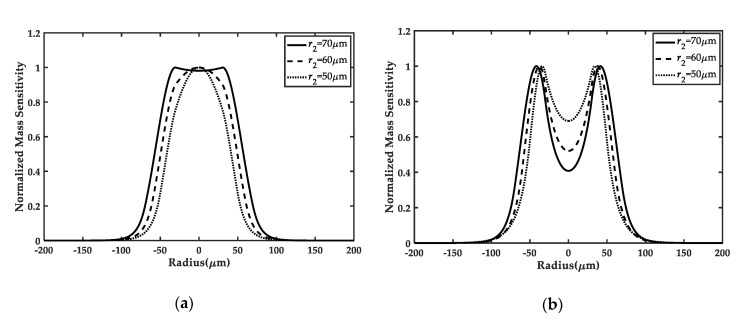
Comparison between the frame-like electrode FBAR (**a**) and the ring electrode FBAR (**b**) [20] for different $r_{2}$, when $r_{1}$ is fixed as 30μm and $\tilde{R}’$ is equal to 0.04. With the increasing of $r_{2}$, more uniform mass sensitivity distributions are observed for the frame-like electrode. However, it is hard for the ring electrode to achieve uniform distributions.

Figure 3a and Figure 4a demonstrate the mass sensitivity distributions along the $x_{1}$ direction of the frame-like electrode FBAR under different inner or outer radii of the electrode, respectively. It can be seen that the uniformity of the mass sensitivity curves in the active area depends on the values of $r_{1}$ and $r_{2}$. For $r_{1}$ with 20μm in Figure 3a and $r_{2}$ with 70μm in Figure 4a, the mass sensitivity distribution of the central region is nearly uniform, which is of benefit to mass sensing. In addition, compared to the ring electrode FBAR in Figure 3b and Figure 4b [20], we can clearly find that the frame-like electrode FBAR has a greater advantage over the reported ring electrode FBAR in achieving the mass sensitivity platform in the active electrode region when the electrode parameters are fixed. Quantitatively, this advantage can also be demonstrated by the mass sensitivity amplitude deviation Δ in Table 1. When Δ gets closer to 0, the mass sensitivity distribution becomes more and more uniform. Particularly, when electrode parameters are selected as $r_{1}=20\;\mu m$, $r_{2}=60\;\mu m$, and $\tilde{R}’=0.04$, the optimization rate $|\Delta_{F}-\Delta_{R}|/\Delta_{R}$ on the uniformity of the mass sensitivity can be up to 97.90%, which is much higher than the optimization rate 77.63% obtained by the reported double-ring electrode design [11]. These results further prove the superiority of the frame-like electrode in achieving uniform mass sensitivity distributions.

**Figure 5:**
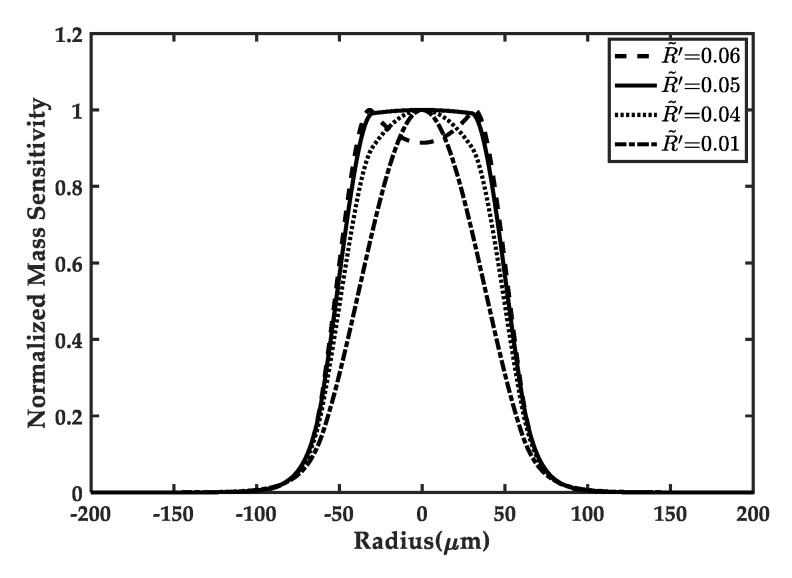
Effects of the mass ratio $\tilde{R}’$ of the overlap electrode to ZnO piezoelectric thin film on the mass sensitivity distributions in the frame-like electrode FBAR sensor, when $r_{1}$ is fixed as 30μm and $r_{2}$ is fixed as 60μm. With the increasing of $\tilde{R}’$, the shape of the distributions change to the concave from convex. The uniform distributions in the active region are obtained for $\tilde{R^{\prime }}=0.05$, indicating that the uniform mass sensitivity distributions can be successfully achieved by selecting proper mass ratio through the novel frame-like electrode design.

In Figure 5, the effect of the overlap driving electrode/ZnO thin film mass ratio $\tilde{R}’$ on mass sensitivity distributions is studied. The frequency *f* and mass sensitivity amplitude deviations *Δ_F_* corresponding to each case are listed in Table 2. It is obvious that the mass ratio can strongly affect the mass sensitivity distributions. With the decreasing of the mass ratio $\tilde{R}’$, the shapes of the mass sensitivity distributions along $x_{1}$ direction alter from a bimodality to a platform and then to a bell-shape, and the deviations *Δ_F_* change from large to small and then to large. Hence, if an appropriate mass ratio is taken in a real structural design, like $\tilde{R}’=0.05$, the difference *Δ_F_* will be closer to zero and a nearly flat mass sensitivity distribution in the active area will be successfully obtained.

**Figure 6:**
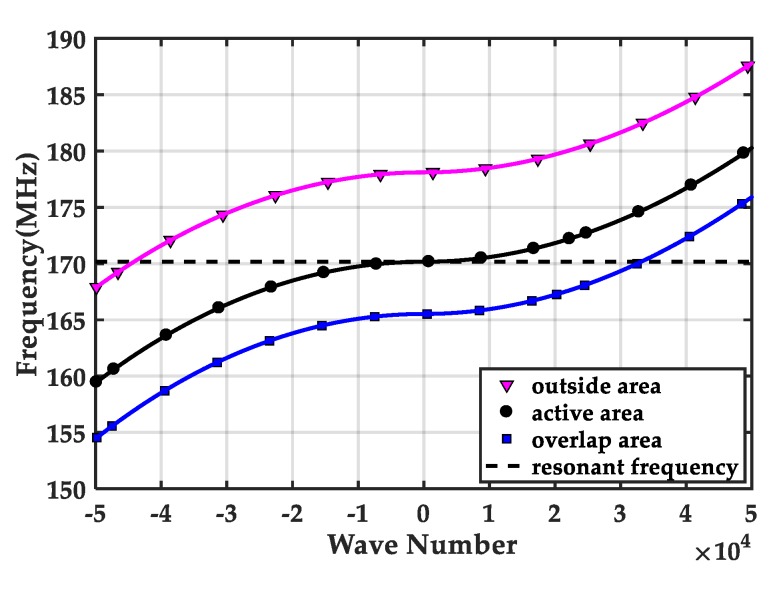
Schematic of the dispersion curve of the thickness extensional (TE) wave modes in the active, overlap, and outside areas.

Lastly, we study the mechanism to achieve a uniform mass sensitivity distribution in the active area from the view of dispersion curve. The dispersion characteristics of the thickness extensional wave near the cut-off frequency for the outside, active, and overlap regions are separately obtained according to Equation (13), as shown in Figure 6. For a pure real ξ, the plate wave can propagate along the in-plane direction. For a pure imaginary ξ, the wave decays exponentially. For a zero wave number, the amplitude of the wave is independent on the in-plane coordinate, resulting in a platform [23]. Therefore, for a fixed resonant frequency, the corresponding mode shape depends on the wave numbers in the outside, overlap, and active area. If the vibration frequency of the whole FBAR mass sensor is equal to the cut-off frequency of the active region, a nearly uniform mass sensitivity platform in the active region can be achieved.

## 5. Conclusions

In this paper, a ZnO FBAR sensor with its top surface covered by a circular frame-like electrode is proposed to achieve a uniform mass sensitivity distribution in its active area. The two-dimensional scalar equations by Tiersten and Steven were employed to study this frame-like electrode FBAR mass sensor, and the resonant frequency and corresponding vibration mode were obtained. The effect of the electrode dimensions and the mass inertia $\tilde{R}’$ were also investigated in detail to achieve a more uniform mass sensitivity distribution. Numerical results show that compared with the reported ring electrode FBAR, our frame-like electrode FBAR can achieve a maximum optimization rate of 97.90% for the uniformity under the same structural parameters, which is much higher than the optimization ratio of 77.63% obtained by the reported double-ring electrode design. The mechanism to achieve the mass sensitivity platform in the active area is clearly stated by analyzing the dispersion curve and proved by the mode shapes under different structural sizes. These results are crucial for the optimization and design of the high-performance FBAR mass sensors. 

## Figures and Tables

**Table 1:** The comparison on the uniformity of mass sensitivity of the frame-like electrode FBAR sensor for different electrode sizes, with ${\tilde{R}}^{\prime }=0.04$ .

*r*_1_(μm)	*r*_2_(μm)	*f* (MHz)	*f_a_* (MHz)	|*f−f_a_*| (MHz)	*Δ*_*F*_	*Δ*_*R*_ [20]	$\frac{\left|\Delta_{F}-\Delta_{R}\right|}{\Delta_{R}}$ (%)
20	60	170.2664	170.1609	0.1055	0.0051	0.2430	97.90
30	60	171.0949	170.1609	0.9340	0.0974	0.3641	73.25
40	60	172.0308	170.1609	1.8699	0.3154	0.4022	21.58
30	50	172.5879	170.1609	2.4270	0.2392	0.2678	10.68
30	60	171.0949	170.1609	0.9340	0.0974	0.3641	73.25
30	70	169.9863	170.1609	0.1746	0.0186	0.3965	95.31

*f* is the resonant frequency of the FBAR mass sensor. *f*_a_ is the cut-off frequency in the active region of the frame-like FBAR. |*f*−*f_a_*| is the deviation between the resonant frequency *f* of the FBAR sensor and the cut-off frequency *f_a_*. *Δ* is the mass sensitivity amplitude deviation between the highest peak and the lowest valley in the active area of FBAR, in which the subscripts F and R stand for the frame-like electrode FBAR and the ring electrode FBAR, respectively. $\frac{\left|\Delta_{F}-\Delta_{R}\right|}{\Delta_{R}}$ is the optimization rate on the uniformity of mass sensitivity distribution in the frame-like electrode FBAR compared with the ring electrode FBAR.

**Table 2:** The comparison on the uniformity of mass sensitivity of the frame-like electrode FBAR sensor for different mass ratios $\tilde{R^{\prime }}$ , with $r_{1}=30\;\mu m$ and $r_{2}=60\;\mu m$.

$\tilde{R^{\prime }}$	*f* (MHz)	*f_a_* (MHz)	|*f* − *f_a_*| (MHz)	*Δ_F_*
0.01	173.4852	170.1609	3.3243	0.3166
0.04	171.0949	170.1609	0.9340	0.0974
0.05	170.2402	170.1609	0.0811	0.0087
0.06	169.3639	170.1609	0.7970	0.0811
